# Zebrafish Shoaling, Its Behavioral and Neurobiological Mechanisms, and Its Alteration by Embryonic Alcohol Exposure: A Review

**DOI:** 10.3389/fnbeh.2020.572175

**Published:** 2020-09-25

**Authors:** Amanda Facciol, Robert Gerlai

**Affiliations:** ^1^Department of Cell and Systems Biology, University of Toronto, Toronto, ON, Canada; ^2^Department of Psychology, University of Toronto Mississauga, Mississauga, ON, Canada

**Keywords:** dopamine, FASD, glutamate, oxytocin, social behavior, zebrafish, shoaling

## Abstract

Social cognition and social behaviors are complex phenomena that involve numerous brain areas and underlying neurobiological mechanisms. Embryonic alcohol exposure may lead to the development of Fetal Alcohol Spectrum Disorder (FASD), a disorder that manifests with varying symptoms including abnormal social behavior and other cognitive deficits. Animal models have been utilized to mimic aspects of the disease and to study potential underlying mechanisms. The zebrafish is a relative newcomer in this field but has been suggested as an optimal compromise between system complexity and practical simplicity for modeling FASD. Importantly, due to external fertilization and development of the embryo outside the mother and subsequent lack of parental care, this species allows precise control of the timing and dose of alcohol delivery during embryonic development. Furthermore, the zebrafish is a highly social species and thus may be particularly appropriate for the analysis of embryonic alcohol-induced alterations in this context. Here, we provide a succinct review focusing on shoaling, a prominent form of social behavior, in zebrafish. We summarize what is known about its behavioral mechanisms and underlying neurobiological processes, and how it is altered by exposure to ethanol during embryonic development. Lastly, we briefly consider possible future directions of research that would help us better understand the relationship between the behavioral expression and molecular basis of embryonic ethanol-induced social deficits in fish and humans.

## Introduction

Social interaction and the expression of social cognition are some of the most essential yet complex behaviors in the animal kingdom. From insects through fish to mammals, social cooperation is often necessary for survival. Social behavior can be expressed in many contexts, including foraging, aggression, competition, and mating. The study of social behavior spans multiple disciplines, including ecology, evolutionary biology, ethology, psychology, pharmacology, and neuroscience (O’Connell and Hofmann, 2011). Studying social interactions in the wild, as is usually conducted in ecology and ethology studies, can give us important insights into key mechanisms and dynamics of social behavior, but understanding what underlies social dysfunction may be equally important and maybe best studied in the laboratory. Such laboratory analyses may be employed for modeling and mechanistic analyses of human central nervous system (CNS) disorders whose core symptoms include abnormalities in social behavior. This review article is centered around such a biomedical focus: modeling fetal alcohol spectrum disorders (FASD) using a relatively underutilized and novel vertebrate laboratory species, the zebrafish.

Several human disorders, including neurodevelopmental disorders like Autism Spectrum Disorders (ASD), neuropsychiatric disorders like schizophrenia, and drug abuse-related disorders like FASD are associated with social dysfunction (Couture et al., 2010; Brüne et al., 2011; Stevens et al., 2015; Fyre, 2018). We can gain information about these disorders from behavioral observation and post-mortem analysis of human patients, but animal models provide substantial advantages for understanding the mechanisms underlying the expression of both natural and dysfunctional social behaviors in a living organism (O’Connell and Hofmann, 2011, 2012). The most popular laboratory species for studying social behavior are rodents, particularly rats and mice. The favorites of genetics, pharmacology, behavioral neuroscience, and developmental biology, these species have been the primary tool for studying social behavior for many years (Phelps et al., 2010; Beery and Kaufer, 2015). The preference for these species has been warranted not only because of their sophisticated behavioral repertoire, including complex social behavior, but also due to decades of research on, and tools and techniques developed for, their genetic, pharmacological, and neurobiological manipulation. Although rodents still dominate in biomedical research, other model organisms are also gaining a foothold. Drosophila (Sokolowski, 2010) and zebrafish (Wright et al., 2006; Oliveira, 2012) have gained attention in biomedical research in general and in the modeling of human CNS disorders in particular.

This review article focuses on the zebrafish, a vertebrate with numerous evolutionarily conserved features shared with mammalian species, including our own. The advantages of zebrafish in several research fields, including psychopharmacology and behavioral neuroscience, have been reviewed extensively (Gerlai et al., 2000; Spence et al., 2007; Norton and Bally-Cuif, 2010; Gerlai, 2011; Stewart et al., 2011). In this review article, we limit our discussion on the use of this species in FASD research. FASD results from the human fetus being exposed to alcohol. The severity and symptomatology of FASD have been known to vary greatly (Manning and Hoyme, 2007), but recent evidence suggests that the cases share a common feature: altered social behavior, i.e., abnormal social cognition (reviewed by Stevens et al., 2015). For example, FASD patients in their childhood exhibit deficient attachment behaviors, during adolescence, they also show a variety of social behavior related deficits, and during adulthood, they are often found to suffer from social withdrawal, depression, and, for example, exhibit aberrant sexual behavior and fail to care for their children (Kelly et al., 2000). Equivalent or similar behavioral alterations have been documented in rodent models of FASD too (Kelly et al., 2000). Rodents, due to their high evolutionary relatedness to human, and because of the similarities in how they and humans respond to embryonic alcohol exposure, have thus become the main subjects of translational biomedical research in the analysis of FASD. How could the zebrafish advance our knowledge in this research area? Why would anyone prefer the zebrafish to rodents in modeling and studying FASD?

This brief review article will attempt to answer these questions pointing out both the advantages and the disadvantages of the zebrafish. We start the review by briefly introducing the human disorder, FASD, followed by discussing the question of face validity of the zebrafish FASD model, i.e., the phenotype expected to be altered by embryonic alcohol exposure. In this context, we focus on a form of social behavior, shoaling (group forming), and review what is known about it in zebrafish. Subsequently, we discuss how embryonic alcohol exposure alters shoaling in zebrafish and why we think focusing on shoaling is warranted in this context. But, at the same time, we also note why such a focus may be insufficient in the analysis of embryonic alcohol-induced changes in zebrafish. Next, we review questions of the construct validity of the zebrafish FASD model, i.e., review the pioneering, and often quite preliminary, findings of potential mechanisms underlying embryonic alcohol exposure-induced social behavior deficits in zebrafish. Last, we delineate some of the future directions of research we find particularly important in the analysis of FASD using the zebrafish.

## Fetal Alcohol Spectrum Disorders (FASD): Why Should We Study Them?

Social dysfunction is present in several human CNS disorders, alcohol-related disorders being only one class of them. Nevertheless, alcohol-related disorders are some of the most common yet debilitating disorders worldwide. Alcohol (ethanol, ethyl alcohol, or EtOH) represents the fifth leading contributor to disability and is globally responsible for more than 5% of deaths (Lim et al., 2012). One particular alcohol-related disorder that is gaining attention is FASD. FASD, which is the most prevalent preventable form of mental disability, arises when a pregnant mother consumes alcohol (Clark and Gibbard, 2013). This consumption exposes the developing fetus to the teratogenic effects of alcohol, which can significantly alter normal embryonic development. Common symptoms in the more severe forms of FASD include morphological deficits (i.e., small eyes, smooth thin philtrum), but even in the milder forms of the disease significant behavioral and cognitive abnormalities have been identified, including learning deficits, social behavior problems, as well as drug addiction (Jones et al., 1973; Streissguth et al., 1978; Sokol et al., 2003). Despite the well-known risks of drinking alcohol while pregnant, as many as 10% of women admit to having continued alcohol consumption during their pregnancy (McHugh et al., 2014; Lange et al., 2016).

FASD is a life-long disease. Patients suffering from the more severe forms require long-term care, which places a large financial burden on society and those suffering from FASD. If alcoholic parents are deemed unfit to care for the child, foster care resources are essential. Children often require special treatment in schools for intellectual disabilities. As adults, extended health care, mental health resources, and substance abuse services may be necessary (Popova et al., 2016). With all these considered, the yearly cost of FASD in Canada alone is estimated to be over $1.8 billion (Popova et al., 2016). There is no cure or appropriate treatment developed for FASD. Even the diagnosis of this disease represents a challenge. The two main reasons for this are, one, we do not understand the mechanisms underlying embryonic alcohol-induced changes, and two, FASD is a highly variable disease. Significant variation in FASD is seen both in the types and severity of symptoms. Underlying this variance are numerous factors, including duration and frequency of drinking during pregnancy, maternal physiology, as well as the developmental stage at which the fetus is exposed to alcohol (Burd and Martsolf, 1989; May et al., 2013). This variation led to the use of the diagnostic umbrella term FASD, under which cases are categorized based upon severity (Burd and Martsolf, 1989; Chudley et al., 2005). FASD encompasses Fetal Alcohol Syndrome (FAS, arising from the most extreme cases of maternal drinking), partial FAS, alcohol-related neurological defects (ARND), and alcohol-related birth defects (ARBD; Landgraf et al., 2013). The less severe cases are the most prevalent and, due to their mild nature, are often un- or misdiagnosed. Despite the varying severity and types of symptoms observed in FASD patients, as mentioned above, a phenotypical abnormality common to most FASD cases has started to be recognized: impaired social behavior.

To increase our understanding of FASD, numerous animal models have been developed (Patten et al., 2014). We have developed an animal model using zebrafish. Given the high prevalence of the milder forms of FASD, we have decided to focus on this end of the spectrum of FASD. In this review article, we discuss this model, focussing first on the behavioral phenotype, shoaling behavior, and subsequently discussing preliminary results about mechanisms of shoaling and embryonic alcohol-induced changes in this species.

## Zebrafish Shoaling Behavior

There are a large number of behaviors classified under social behavior, including courtship and sexual behaviors, aggression (agonistic interactions), parental behaviors, and other forms of affiliative behaviors including group forming (social cohesion). As mentioned above, several of these behaviors have been found impaired or abnormal in FASD patients, and have been also found altered in rodent models of FASD (Kelly et al., 2000). However, a PubMed search with terms (“FASD” OR “embryonic alcohol”) AND “zebrafish” AND (“sexual behavior” OR “courtship” OR “aggression”) returns no published studies as of the writing of this review article. That is, zebrafish have not been used in such analyses, perhaps because these respective behaviors have not been well characterized in this species. Nevertheless, there is one behavior, shoaling, which is altered by embryonic alcohol exposure in zebrafish, the focus of this review article (Fernandes and Gerlai, 2009).

Shoaling is a prominent feature of the zebrafish. It is the highly social nature of this species that experimenters argue makes the zebrafish particularly useful for the understanding of the complexity of social behaviors and underlying biological mechanisms (Oliveira, 2012, 2013).

In nature, zebrafish aggregate and form social groups, called “shoals.” The dynamics of this group forming behavior is highly complex and debated to this day. Numerous environmental factors play roles in shoal formation, including vegetation, water flow speed, temperature, age of the fish, geographical location, existing shoal size, predation, early life experience, and food availability, to name a few (Ruhl and McRobert, 2005; Miller and Gerlai, 2007, 2011; Arganda et al., 2012; Suriyampola et al., 2016; Orger and de Polavieja, 2017; Groneberg et al., 2020). For example, zebrafish in still waters are more likely to form smaller shoals compared to fast-moving water, where shoals of upwards of 300 individuals have been observed (Suriyampola et al., 2016).

Shoaling confers several adaptive advantages by increasing foraging efficiency and predator avoidance. Having a large number of individuals together means the group is more vigilant and can detect the presence of predators more efficiently (“many eyes” hypothesis; Pitcher and Parrish, 1993), is better able to detect food, and the fish in the group are more likely to escape in the event of an attack, termed the dilution effect. Swimming as a group also represents a hydrodynamic advantage, whereby the individuals swimming at the back use less energy due to reduced water resistance (Pitcher and Parrish, 1993; Krause and Ruxton, 2002; Miller and Gerlai, 2011; Suriyampola et al., 2016).

Conversely, shoaling also comes with several disadvantages. Many individuals being close to each other can result in the quicker transfer of pathogens and higher levels of competition for both food and mates. Also, large groups of fish are more easily spotted by predators compared to a single fish that may be able to hide in vegetation (Pitcher and Parrish, 1993; Krause and Ruxton, 2002; Miller and Gerlai, 2011; Suriyampola et al., 2016). The position of an individual within a shoal is also a key factor to consider when weighing advantages vs. disadvantages. For example, an individual at the perimeter of the shoal will have quicker access to food but will be more susceptible to predation. On the other hand, those in the middle are more protected from predatory attacks but will have limited access to food (Bumann et al., 1997; Miller and Gerlai, 2011). Taken together, the art of shoaling represents a delicate balance between safety from predation and access to resources, a balance that may explain the observed short-time scale oscillations of shoal cohesion described previously (Miller and Gerlai, 2007). In summary, shoaling is a fascinating and robust phenotypical feature of zebrafish underlying of which there may be complex cognitive processes. How similar these processes may be across fish and mammals, and whether alterations of these processes by embryonic alcohol exposure in the zebrafish mimic at least some aspects of human FASD are questions to which we return later when we discuss the mechanisms underlying FASD. But first, we consider another question, the development of shoaling in zebrafish.

### The Development of Shoaling Behavior

Embryonic alcohol exposure has been shown to alter shoaling in adult zebrafish (Fernandes and Gerlai, 2009), a lasting change that is observable even in old zebrafish, i.e., at 2 years of age (Fernandes et al., 2015b). Could embryonic alcohol exposure induced alterations to be detected earlier, i.e., soon after the fish become free swimming at age 5 days post-fertilization (dpf)? This question may be answered if we understand the development of shoaling in zebrafish, the focus of this brief section.

Surprisingly, only recently has the development of shoaling started to be studied in zebrafish (Buske and Gerlai, 2011a). After fertilization, zebrafish remain in their eggs for approximately 2–3 dpf (Kimmel et al., 1995) and do not shoal immediately after hatching. For the first few days post-hatching, young larval zebrafish are quite immobile, adhering to the walls and floor of the tank and only begin to swim freely at approximately 5–6 dpf (Engeszer et al., 2007; Hinz and de Polavieja, 2017). While one study suggests social preference may arise as early as 6–8 dpf (Hinz et al., 2013), a shoaling response that is statistically distinguishable from random distribution can only be observed after 2 weeks of age (14–15 dpf; Engeszer et al., 2007; Buske and Gerlai, 2011a; Dreosti et al., 2015; Hinz and de Polavieja, 2017).

Social preference and shoaling responses in adult zebrafish are highly dependent on early life experience. A social preference experiment by Engeszer et al. (2004) demonstrated that when striped wildtype (WT) zebrafish and non-striped *nacre* zebrafish (lacking stripes due to mutation in the *mitfa* gene) were reared either with their genotype or the opposite (WT raised with *nacre* and vice versa), the experimental fish preferred the phenotype they were reared with, regardless of their genotype. Lastly, zebrafish raised in complete social isolation were found to stay significantly further away from shoal mates and showed diminished social preference compared to zebrafish raised in normal social conditions, demonstrating that lack of access to social stimuli during early development leads to abnormal social response (Engeszer et al., 2004; Shams et al., 2018; Groneberg et al., 2020). Preliminary studies of our own also suggest that enriched social and/or physical environment may rescue embryonic alcohol exposure-induced social behavioral deficits in zebrafish. However, irrespective of how well and how early social deficits may be measured in the developing zebrafish, given the transparent nature of this fish for the first several days of its life, mechanistic alterations in brain development induced by embryonic alcohol exposure may be most easily uncovered and followed in the zebrafish, a feat that would be impossible with rodents, or in fact with any vertebrate organisms other than fish.

### Dynamics Within a Shoal

For proper analysis of alcohol-induced alterations in shoaling, or the effects of other experimental manipulations on this behavior, one may need to understand the complex interactions among zebrafish within the shoal that leads to the stable formation of the group, the traffic rules of shoaling. Many factors influence the dynamics of movement within a shoal. However, we must first make the distinction between “shoaling” and “schooling.” In a shoal, zebrafish aggregate together, respond to each other’s movements, and keep a fairly steady distance from each other, but do not necessarily move in unison. Schooling, on the other hand, represents synchronized movements (Pitcher and Parrish, 1993), a long-held belief that was first experimentally/statistically proven by Miller and Gerlai (2012). While zebrafish spend more time shoaling than schooling, certain environmental factors, such as stress levels and group size, can trigger short term schooling responses in adult zebrafish (Miller and Gerlai, 2011). By now we also know that acute alcohol and nicotine have distinct effects on shoal dynamics, the former particularly affecting polarization of movement (Miller et al., 2013).

The strength of shoal cohesion (i.e., how close fish are together) is also dependent upon environmental factors. Stress-inducing situations, such as a new environment or the approach of a predator, often result in tighter shoal cohesion, which eventually loosens as the fish habituate to the environment (Hoare et al., 2004; Miller and Gerlai, 2011). The effect of stress and anxiety on shoaling behavior is an important observation from the perspective of FASD research because embryonic alcohol exposure has been suspected to alter stress and anxiety in humans (e.g., Temple et al., 2019) and zebrafish alike (Baiamonte et al., 2015), a question we will return later.

Lastly, we note that social interaction between individuals is not unidirectional. Zebrafish’s social responses are highly dependent upon the behavior of the other individuals. Although this has not been studied in a natural setting, one-on-one social interaction has been analyzed in the laboratory. For example, Stednitz et al. (2018) found pairs of experimental zebrafish to “orient” toward each other, whereby they not only move close together, but they face each other and swim in a coordinated fashion (Stednitz et al., 2018). If this social orienting behavior were to be eliminated in one of the zebrafish (*via* brain lesioning or administration of an NMDA receptor antagonist, MK-801), the non-treated zebrafish was found to respond with diminished orientation (Stednitz et al., 2018; de Polavieja and Orger, 2018). This individual-level social interaction analysis may be highly useful for detecting fine-grained effects of embryonic alcohol exposure in zebrafish, a hypothesis that has not yet been tested empirically.

### Quantification of Zebrafish Shoaling and Social Preference in the Laboratory

Proper quantification of shoaling and schooling, as demonstrated by the above-discussed studies, is a must for the evaluation of the effects of embryonic alcohol exposure in zebrafish. Most of what we know about zebrafish social behavior comes from laboratory studies (Suriyampola et al., 2016). Two distinct types of experimental paradigms exist to investigate shoaling behavior in zebrafish. The first type involves placing multiple fish into one large experimental tank and measuring, for example, the inter-individual distance between shoal mates (Miller and Gerlai, 2007; Saverino and Gerlai, 2008; Buske and Gerlai, 2011a). This testing set up is useful for understanding shoal dynamics under natural or semi-natural conditions, and, for example, already allowed investigators to distinguish the acute effects of commonly abused drugs, including nicotine and alcohol (Miller et al., 2013). Nevertheless, analyzing shoaling behavior in this manner has limited utility when it is employed to study artificially induced social deficits, such as those resulting from exposure to alcohol during embryonic development. Similarly, proper characterization of the effects of mutations that may alter the effects of alcohol may also be complicated using the free-swimming shoal analysis approach. For example, identification of mutant outliers necessitates measuring the behavior of single experimental subjects. In a free-swimming shoal, the single experimental individual must be tagged or marked to visually distinguish it from the others, a goal that is possible to accomplish, e.g., with subcutaneous injection of colored dye (Cheung et al., 2013), although some successful attempts to address individual tracking have been made using software solutions too. However, irrespective of whether such marking methods are invasive or non-invasive, in a freely moving shoal, or even in the case of two interacting subjects, it is difficult to deduce whether the difference in behavior is indeed due to alteration of the experimental fish, or the modified responses of shoal-mates towards it.

The second type of social paradigm solves the above problem. It involves placing a single individual into a tank and presenting conspecifics or stimuli resembling conspecifics to this subject (Fernandes et al., 2015a,b; Zimmermann et al., 2016; Shams et al., 2018; Landin et al., 2020). In this situation, behaviors such as distance to stimulus and duration spent near the stimulus are used to quantify the strength of social behavior. In this type of experiment, both live and animated conspecifics have been used successfully (Saverino and Gerlai, 2008; Qin et al., 2014). Interestingly, previous studies have demonstrated that zebrafish respond similarly, both behaviorally and neurochemically, to animated and live social stimuli (Qin et al., 2014; Shams et al., 2018). These studies suggested that interactions between the stimulus and the experimental zebrafish may not be required, and even three-dimensional movement may not be necessary for the stimulus to induce a maximal shoaling response in the experimental fish. Larsch and Baier (2018), using animated (moving) dots also concluded that reciprocal interaction is not required and realistic movement patterns simulating live zebrafish swimming are enough to induce affiliative responses in juvenile zebrafish.

Furthermore, certain modifications to the animated images of zebrafish, i.e., change in color, pattern, size, moving speed, etc., have also been found to alter the response of experimental zebrafish (e.g., Saverino and Gerlai, 2008). The ability to precisely control the visual features of the animated stimuli, however, comes with a price. The animated stimulus fish do not interact with the experimental subject, which some view as an excellent and consistent control, while others may criticize it as unnatural. Also, animated images do not provide multimodal stimuli, e.g., auditory, olfactory and lateral line cues, a fish may perceive in a natural freely moving shoal. This lack of multimodal cues is also an issue with live stimulus fish that are usually presented in a tank outside of the experimental tank of the test fish. The modality of social stimuli is not just an academic question. Fernandes et al. (2019) found the social deficits of juvenile zebrafish exposed to alcohol during their embryonic development to be dependent upon non-visual social cues. When visual social cues were present, the social deficit was not observable.

### Neural Mechanisms of Shoaling Behavior

The cornerstone of zebrafish FASD models is that they promise translational relevance (Seguin and Gerlai, 2017). However, in the context of social behavior in general, and shoaling in particular, translational relevance may be ascertained only if the mechanisms underlying the zebrafish behavior are evolutionarily conserved, i.e., if they overlap with those of some of the human social behaviors. Unfortunately, however, not enough is known about the mechanisms of zebrafish social behaviors, and thus the question of evolutionary conservation, and along with it, the question of the construct validity of zebrafish FASD models remains mainly unanswered. Nevertheless, pioneering studies offer promising results. Here, we consider two mechanistic similarities between mammals and zebrafish, one at the level of gross anatomy, and another at a molecular/neurotransmitter level, as some of the first examples of translational relevance for zebrafish in the contexts of social behavior and FASD research.

Several mammalian brain areas are involved in mediating aspects of social behavior, and regions homologous to these mammalian structures have been mapped in the zebrafish brain (Geng and Peterson, 2019). For example, areas such as the amygdala (social recognition and emotion), hippocampus (e.g., social learning), nucleus accumbens, and the ventral tegmental area (social reward) in mammals are homologous to the zebrafish ventral telencephalon, lateral dorsal telencephalon, dorsal-ventral telencephalon, and the posterior tuberculum, respectively (Geng and Peterson, 2019). However, whether these brain areas play particular roles in the alterations induced by embryonic alcohol exposure in zebrafish has not been shown.

The dopaminergic and serotonergic systems have been shown to play key roles in the expression of zebrafish social behavior (Fernandes and Gerlai, 2009; Buske and Gerlai, 2012; Fernandes et al., 2015a). Numerous studies have found elevated levels of these neurotransmitters (particularly dopamine) shortly after exposure to conspecifics (Fernandes and Gerlai, 2009; Buske and Gerlai, 2011b, 2012; Saif et al., 2013; Fernandes et al., 2015a; Shams et al., 2018). Pharmacological disruption of these systems, *via* receptor antagonists (e.g., a D1 dopamine receptor antagonist), results in abnormal expression of social behavior (Scerbina et al., 2012). Furthermore, there is strong evidence that embryonic alcohol exposure does alter the functioning of these neurotransmitter systems in zebrafish (Buske and Gerlai, 2011b; Fernandes et al., 2015a) similarly to mammals (e.g., Shen et al., 1999), a question, we will return later.

Lastly, isotocin, the fish equivalent of the mammalian neurohormone oxytocin, has been found to play a key, yet complex, role in the expression of social behavior. Oxytocin is known to play major roles in a variety of mammalian social behaviors (for a recent review, see Ebert and Brüne, 2018). Administration of oxytocin to adult zebrafish results in a dose-dependent increase of social preference and a decrease of fear (Braida et al., 2012). Additionally, a study by Landin et al. (2020) showed that administration of the oxytocin antagonist, L-368-889, decreased adult zebrafish social behavior, manifesting as a larger distance between the test fish and the social stimulus. The question of whether the isotocin system is altered by embryonic alcohol exposure in zebrafish, however, has not been answered.

In summary, the above-cited and other pioneering studies have started to unravel the likely complex biochemical and neuroanatomical networks mediating social behaviors in zebrafish, but the questions regarding evolutionary conservation of these mechanisms and whether they represent translationally relevant processes overlapping between human FASD and zebrafish FASD models are yet to be addressed.

## Zebrafish FASD Models

While rodents are the leading animal model for most alcohol-related studies, zebrafish are becoming highly popular for modeling FASD (Patten et al., 2014). There are numerous reasons for this. The small size, simple husbandry, ease of drug administration, and external fertilization of zebrafish make them highly advantageous for this field of work (see reviews Ali et al., 2011b; Lovely et al., 2016; Fernandes et al., 2018; Seguin and Gerlai, 2018; Facciol et al., 2019). The behavioral deficits observed in humans suffering from FASD map nicely onto the zebrafish behavioral repertoire, including social behavior, learning, anxiety, and addictive/withdrawal behaviors (Gerlai et al., 2000; Buske and Gerlai, 2011b; Kalueff et al., 2013; Sterling et al., 2015; Gerlai, 2016). Additionally, as mentioned above the zebrafish has evolutionarily well-conserved biological systems, where particular neurochemical pathways and neural structures in mammals have homologs in the zebrafish (Kalueff et al., 2014; Horzmann and Freeman, 2016; Geng and Peterson, 2019). Yet, the zebrafish is simpler, and it is small, easy to breed and keep in large numbers, and thus offers an excellent reductionist approach.

Previous FASD studies in zebrafish commonly administered higher doses of ethanol for extended periods (Carvan et al., 2004; Arenzana et al., 2006; Ali et al., 2011a; Parker et al., 2014). However, this dose and duration may not be representative of how typical pregnant women would drink. Therefore, more focus is now being placed on modeling the milder forms of FASD. While this milder dosing regime has been shown to not increase mortality or result in morphological deficits, there is evidence for significant behavioral abnormalities. Embryonic ethanol-induced deficits in learning and memory (Carvan et al., 2004; Fernandes et al., 2014), anxiety (Parker et al., 2014; Bailey et al., 2015), social response (Fernandes and Gerlai, 2009; Buske and Gerlai, 2011b; Parker et al., 2014; Fernandes et al., 2015a,b), and even alcohol self-administration (Sterling et al., 2016) have been successfully modeled in zebrafish. Given that shoaling (a form of social behavior) is a highly prominent feature of the zebrafish, and given the complexity of FASD-related social deficits in humans, there is an increasing focus on studying embryonic ethanol-induced shoaling behavior deficits in zebrafish. Here, we will summarize what is currently known about how embryonic ethanol exposure in zebrafish alters this form of social behavior, from both a behavioral and underlying mechanisms perspective, with a focus on modeling the mild cases of embryonic ethanol exposure.

### FASD and Zebrafish Shoaling Behavior

An in-depth summary of how embryonic ethanol exposure alters behavioral responses in zebrafish can be found elsewhere (Ali et al., 2011b; Lovely et al., 2016; Fernandes et al., 2018; Seguin and Gerlai, 2018; Facciol et al., 2019). Briefly, the main result is that exposure to low doses of ethanol (between 0.25% and 1% vol/vol) for only 2 h is enough to significantly alter shoaling. This social dysfunction is dose-dependent, with higher doses resulting in greater shoaling behavior deficit (Fernandes and Gerlai, 2009). Importantly, deficits in shoaling have been found with exposure to doses as low as 0.12% vol/vol ethanol (Parker et al., 2014). This highlights the fact that even the mildest forms of maternal drinking can have serious repercussions for offspring. The deficit manifests as increased shoal distance (decreased shoal cohesion) in freely moving shoals of zebrafish (Buske and Gerlai, 2011b), as well as the increased distance between the single experimental fish and animated images of conspecifics (Fernandes and Gerlai, 2009). Notably, the impairment that is induced by a 2 h long alcohol exposure at 24 h post-fertilization (hpf) is detectable even at 2 years of age in the exposed fish (Fernandes et al., 2015b). Furthermore, the impairment induced by this particular alcohol exposure regimen was found not to be accompanied by abnormal motor function, perception, or fear/anxiety responses, and is claimed to be specific to social behavior (Fernandes and Gerlai, 2009; Buske and Gerlai, 2011b; Fernandes et al., 2015b; Seguin et al., 2016). However, whether the social behavior deficit is specific or limited to shoaling/group forming in zebrafish, has not been studied.

### Mechanisms Underlying Embryonic Ethanol-Induced Shoaling Behavior Deficits

At the molecular level, ethanol-induced dysfunction in several neurotransmitter systems has been thought to underlie the observed social behavioral deficits. Particularly, low ethanol doses administered during early development has been found to alter both the dopaminergic and serotonergic systems, an effect observed as early as 7 dpf, which lasts well into adulthood (Buske and Gerlai, 2011b; Parker et al., 2014; Fernandes et al., 2015a; Facciol et al., 2020). In the serotonergic system, larval zebrafish exposed to 0.12% (vol/vol) ethanol showed increased expression of *slc6a4* (a serotonin transporter gene) and *htr1aa* (a serotonin receptor gene) at approximately 60 hpf, followed by a decrease in expression of *htr1aa* by 7 dpf (Parker et al., 2014). At 30 dpf, juvenile zebrafish previously exposed to 1% ethanol during early development had lower levels of both dopamine and serotonin compared to non-exposed zebrafish, depending on the developmental stage of exposure (Facciol et al., 2020). Similar deficits in the dopaminergic and serotonergic system are also observed in adults previously exposed to embryonic ethanol (Buske and Gerlai, 2011b; Fernandes et al., 2015a).

It is also notable that the reduced dopamine levels did not seem to be the result of impairments in baseline dopamine synthesis or dopaminergic neurotransmitter system function as unstimulated, socially isolated zebrafish exposed to embryonic alcohol exhibited dopamine and DOPAC levels statistically indistinguishable from those of alcohol unexposed control fish. However, upon delivery of social stimuli, the control fish responded with a rapid elevation of dopamine and DOPAC, but the embryonic alcohol-exposed fish did not (Fernandes et al., 2015b). These results led us to speculate that perhaps the dopaminergic system itself is not impaired, but rather, other pathways or mechanisms in the brain that would stimulate this neurotransmitter system may be affected (Gerlai, 2020a). This hypothesis, i.e., the lack of dopaminergic neurotransmitter system-specific effects of embryonic alcohol exposure, is in line with recent findings demonstrating robust and widespread changes in the brain of adult zebrafish that were exposed to alcohol during their embryonic development. These changes included reduced levels of proteins involved in neuronal plasticity, synaptic transmission as well as brain development, such as brain-derived neurotrophic factor (BDNF), neuronal cell adhesion molecule (NCAM), and the synaptic protein synaptophysin (Mahabir et al., 2018). In addition to such neuron-specific phenotypes, numerous glial cell-related phenotypical alterations, including in astrocytes and oligodendrocytes, have also been detected in the embryonic alcohol-exposed fish in a range of brain areas studied (Chatterjee et al., 2020). Thus, the idea that only very specific brain areas, e.g., those where dopaminergic neurons reside, are affected by embryonic alcohol exposure is highly unlikely. Nevertheless, the question of whether brain areas homologous to mammalian structures known to be involved in social behaviors are specifically or selectively affected by embryonic alcohol exposure in zebrafish has not been systematically explored.

In summary, although the behavioral effects of being exposed to only low levels of alcohol during embryonic development appear to be fairly specific to complex cognitive functions like learning and memory, and social behavior, the underlying mechanisms are likely widespread, complex, and involve alterations in both neuronal and glial cell function. Furthermore, how well these preliminary mechanistic findings correspond to neurobiological alterations in human FASD will need to be addressed in the future.

## Future Directions

Our current understanding of the evolutionary origin and adaptive nature of social behavior, like shoaling, in fish is rudimentary. Similarly, both the cognitive and the neurobiological mechanisms underlying or mediating shoaling and other forms of social behavior in fish are also not well understood. Furthermore, and for the above reasons too, causal relationships among social behavioral deficits and neurobiological changes detected in response to embryonic alcohol exposure in zebrafish have yet to be established. The zebrafish, with its relative simplicity among vertebrates, but with the arsenal of molecular and other neuroscience tools developed for it, is positioned well for being one of the best vertebrate laboratory organisms with which such questions may be addressed. Listing up all the future research avenues one may follow from where we are today would take volumes. Instead, we only sample a few, a short-list based upon our own biases.

Although the adaptive significance of shoaling may seem to be a tangential question from the biomedical research perspective or the question of translational relevance, we start our list of future directions with this question, because we think it is important both from basic research as well as from the clinical perspective. Although other species have been used for ecology and ethology research in the context of social behavior, and although likely findings of these studies translate well to zebrafish, only very rarely zebrafish have been observed in nature and thus we know little about the natural selection forces that may have shaped shoaling in this species. We also know little about how shoaling behavior is affected by abiotic and biotic characteristics of the natural environment of zebrafish. Gaining information on these questions will help us design our laboratory studies better, an important point that has been discussed in the context of mouse neurobehavioral genetic research (Gerlai and Clayton, 1999; Gerlai, 2002), but one which is equally valid for studies of FASD with zebrafish.

Another particularly important future research area is cognitive mechanisms. Shoaling requires the subject to monitor a potentially large number of shoal-mates, their movements, their status, perhaps dominance status, and numerous subtle physical characteristics of these shoal-mates. This is a highly cognitively demanding task, and we do not know what aspects of the shoal-mates zebrafish evaluate, and what cognitive limits of the demands of such tasks may exist for zebrafish. However, we do know, for example, that zebrafish, similarly to other fish species, as well as other vertebrate species too, can distinguish shoals based upon the number of shoal members (Seguin and Gerlai, 2017). From this initial observation, numerous follow-up studies may be envisioned that could explore the behavioral mechanisms upon which zebrafish base their decision to choose or abandon particular shoals, similarly to how quantity discrimination abilities have been explored with another fish species, the freshwater angelfish (see the study by Gómez-Laplaza and Gerlai, 2020 in this special issue). Furthermore, although some pioneering studies have been conducted on the subject (e.g., Saverino and Gerlai, 2008), we know very little about how zebrafish determine who is a conspecific and who is a hetero-specific fish. Systematically manipulating the visual features of conspecific images will allow the investigator to discover fine-grained deficiencies in social behavioral responses induced by embryonic alcohol exposure in zebrafish.

Shoal dynamics: We also do not clearly understand the traffic rules within the shoal. Even such a fundamental question as to how zebrafish establish a steady inter-individual distance with other shoal members is not clearly understood, although we do know that this distance is usually about 4 body lengths for adult zebrafish (Miller and Gerlai, 2007), and we know that shoaling is not dependent purely on visual cues, but can be performed also using lateral line cues (for review and examples see Miller and Gerlai, 2011). We also do not know how the shoal decides to move or stay, i.e., whether to shoal or school (Miller and Gerlai, 2012), and we do not know whether there are stable leaders and followers or other stable shoal behavior strategies particular shoal members exhibit. Properly characterizing the complexities of shoaling behavior will give us a better handle on behavioral experimentation and thus will allow us a better resolution characterization of the social deficits induced by embryonic alcohol exposure in zebrafish. In addition to the analysis of shoaling behavior, the question of cognitive performance, strongly tied to social cognition, may also be studied in systematic analyses of learning and memory. However, only very few studies have characterized mnemonic and cognitive features of the zebrafish (for recent reviews, see Gerlai, 2016, 2020b), and thus this domain of research must also be strengthened in the future.

The last future research direction within the domain of behavioral analysis concerns a systematic screen for embryonic alcohol-induced behavioral changes. Although in our research, and in research conducted by others too, numerous behavioral features have been tested as possible endpoints of embryonic alcohol exposure in zebrafish, and although among these studies the deficits are seen in shoals as well as in learning clearly stand out, no systematic behavioral analysis using test batteries have been performed in this context.

Mechanistic research into the cognitive processes underlying shoaling, underlying alterations in shoaling induced by embryonic alcohol exposure, and underlying embryonic alcohol exposure, in general, are even less well developed. The mechanistic analysis will likely require comprehensive screening approaches, e.g., mutagenesis screens or drug screens that could identify molecular targets, and/or systematic transcriptome studies, for example. Alternatively, or also, hypothesis-driven in-depth analyses of numerous neurobiological processes as well as of brain areas may be conducted. An interesting example of possible hypothesis-driven research would be to focus on the isotocin system. Similar to oxytocin in mammals, this neurohormone plays a key role in neurodevelopment as well as in the expression of social behaviors in fish (Gutnick et al., 2011; Biran et al., 2018). Interestingly, the injection of an oxytocin receptor antagonist produces social behavior deficits similar to those observed in zebrafish embryonically exposed to ethanol (Landin et al., 2020). Therefore, isotocin may play an important role in the social deficits resulting from embryonic ethanol exposure. However, very few studies have investigated how isotocin is altered with embryonic ethanol exposure. A study by Parker et al. (2014) found low ethanol doses to result in overexpression of the *oxtr* gene (coding for an oxytocin receptor) as early as 50 hpf, while Coffey et al. (2013) also found increased expression of the *oxtl* gene (oxytocin-like gene) in the hindbrain of 6-day-old zebrafish. These studies present key evidence that isotocin may be involved in FASD-related social deficits, albeit their focus has been limited to larval zebrafish (up to 6 dpf). In rodents, prenatal ethanol (PNE)-induced social deficits have been associated with increased oxytocin receptor binding in early adolescent rats (Holman et al., 2018) as well as decreased oxytocin levels (McMurray et al., 2008). Despite these studies, the link between oxytocin activity and the expression of PNE-induced social deficits remains poorly understood.

Another neurochemical system involved in embryonic alcohol-induced social behavior deficits could be the glutamatergic system. Similarly in humans and rodents, glutamate is the most common excitatory neurotransmitter in the zebrafish brain and is involved in neuronal communication across a complex neuronal network with many different functions, including neurodevelopment, movement, and learning (Horzmann and Freeman, 2016). While glutamate is not the main neurotransmitter that comes to mind concerning social behavior, it is important to remember that learning plays a crucial role in the expression of social behavior, and restricting access to social stimuli during early development has been shown to result in abnormal social behavior in adulthood (Shams et al., 2018; Groneberg et al., 2020). Studies by Maaswinkel et al. (2013) and Dreosti et al. (2015) have shown that administration of MK-801 (an NDMA receptor antagonist) decreased shoaling behavior, an effect that was rescued by administration of the oxytocin agonist, carbetocin (Zimmermann et al., 2016). This lends more support to the idea that it may not be one neurochemical system, but the interaction of multiple systems that results in the expression and/or dysfunction of social behavior. To date, no study to our knowledge has systematically analyzed how embryonic ethanol exposure may alter glutamatergic functioning in zebrafish. In adult humans, post-mortem analysis of the hippocampal formation shows alcoholics having increased expression of NMDA-receptor related genes compared to non-drinking humans, namely in genes encoding the GluN1, GluN2A, GluN2C, and GluN3A subunits of this receptor (Jin et al., 2014). In rodents, PNE has been shown to result in decreased NMDA-R binding in several brain areas (Savage et al., 1991; Honse et al., 2003). However, which receptor subunits play a role in this alteration is still debated. One study showed PNE-induced alteration in NMDA receptor subunits, specifically increased hippocampal levels of GluN1 and GluN3A and decreased levels of GluN2B compared to controls (Brady et al., 2013). While other studies have reported PNE-induced decreases in GluN2B (Toso et al., 2005, 2006), and yet another has reported no effect (Bellinger et al., 2002). Interestingly, overexpression of the gene encoding GluN2B has been shown to enhance social recognition, suggesting this receptor subunit may play an important role in the social deficits associated with PNE (Jacobs and Tsien, 2012; Bird et al., 2015).

Potential neuroanatomical locale-specific embryonic alcohol exposure-induced changes have also not been studied and the question of how well such changes map onto what we know about alterations in the brain of human FASD patients will also need to be addressed. Furthermore, the question of developmentally sensitive periods during which the teratogenic effects of alcohol are particularly devastating have started to be analyzed with zebrafish, and will likely be a highly successful research line in the future. It will likely allow us to gain a detailed mechanistic understanding of why FASD represents such varied symptomatology in humans. We also find it important to emphasize that such mechanistic insights will also likely lead to the establishment of detectable biomarkers that may be used as a diagnostic tool in the human clinic.

Last, we mention an entire field of biology that we deliberately left out from this review up till now, epigenetics. Alcohol is known to alter the activity of DNA methyltransferases causing hypomethylation of promoters of genes, and alcohol also influences histone chemistry thereby causing stable and lasting changes in gene expression (e.g., Shukla et al., 2008). When employed during embryonic development, alcohol thus may set up a robustly altered expressome leading to a cascade of developmental changes as well as lasting modification of brain function that will manifest throughout the life of the affected organism. Systematic analysis of epigenetic changes combined with comprehensive mRNA expression analysis using, for example, modern deep sequencing methods or DNA microarrays (Pan et al., 2011) will likely be a highly fruitful future research avenue.

## Conclusions

Human FASD is completely preventable by abstinence, yet remains a highly prevalent disease, leading to lifelong suffering in the affected patient. Animal modeling of this human disorder is crucial as it may shed light on mechanistic details that otherwise would be difficult to discover. Mechanistic understanding of FASD may be important for the development of proper treatment and even for proper diagnostic tools. Although FASD is a highly variable disease, and although the severity and the type of social behavior abnormalities seen in FASD patients also vary, social behavior impairment is increasingly recognized as one of the hallmarks of FASD. Thus, animal models of FASD that allow quantification of complex social behavior, and alterations in such behavior are likely to be useful. Social interactions present complex cognitive demands leading to behavioral responses that are influenced by several internal and external factors. Research investigating such factors is in its infancy. Shoaling is a prominent feature of the zebrafish, and it represents perhaps the richest source of information about the social cognition abilities of this species. Addressing proximate (mechanistic) and ultimate (evolutionary) questions in this context will likely be fruitful with the zebrafish, a simple vertebrate species. Although simple, and separated from our species by about 400 million years of biological evolution, due to the numerous evolutionarily conserved features of the zebrafish, this species is considered a translationally relevant tool. Evolutionary conservation coupled with biological system simplicity may be an important advantage that zebrafish researchers will be able to exploit in the analysis of vertebrate social cognition in general and in modeling and analysis of human FASD in particular. Given the large number of patients suffering from FASD, studying and modeling this disorder with zebrafish will likely be not only an interesting basic scientific inquiry but also one which will have important consequences for the human clinic.

## Author Contributions

AF and RG conceptualized and wrote the review. All authors contributed to the article and approved the submitted version.

## Conflict of Interest

The authors declare that the research was conducted in the absence of any commercial or financial relationships that could be construed as a potential conflict of interest.
